# Characterization of SH3GLB1 in the auditory system and its potential role in mitophagy

**DOI:** 10.1016/j.gendis.2023.05.017

**Published:** 2023-07-06

**Authors:** Xue Gao, Weiqian Wang, Jincao Xu, Shasha Huang, Kun Yang, Jinyuan Yang, Yijin Chen, Guojian Wang, Mingyu Han, Zhendong Wang, Dongyang Kang, Yongyi Yuan, Pu Dai

**Affiliations:** a College of Otolaryngology Head and Neck Surgery, Chinese PLA General Hospital, Chinese PLA Medical School, Beijing 100853, China; b National Clinical Research Center for Otolaryngologic Diseases, State Key Lab of Hearing Science, Ministry of Education, Beijing 100853, China; c Beijing Key Lab of Hearing Impairment Prevention and Treatment, Chinese PLA Medical School, Beijing 100853, China; d Department of Otolaryngology, PLA Rocket Force Characteristic Medical Center, Beijing 100088, China

Mitochondria energize the inner ear to maintain the cochlear potential created by the stria vascularis, assist the motility of outer hair cells, perform synaptic processes, and maintain the spontaneous and sound-driven discharges of the spiral ganglion neurons (SGNs). Mitophagy deficiencies induce the accumulation of damaged organelles and mitochondria in cells and are a primary cause of drug-induced hearing loss.^1^ SH3GLB1, also known as endophilin B1, encoded by the *SH3GLB1* gene, is a multifunctional protein that controls mitophagy, apoptosis, and autophagy. By influencing mitophagy, SH3GLB1 has been linked to the pathophysiological processes of neurodegenerative disorders, including Alzheimer's and Parkinson's disease.^2^ The role of SH3GLB1 in hearing is currently unclear. This study shows the localization of Sh3glb1 in the mouse inner ear, especially in SGNs and inner hair cells (IHCs), suggesting a role in auditory function. The *sh3glb1a* morpholino knockdown zebrafish demonstrated a considerable reduction in inner ear hair cells and neuromasts accompanied by malformation of the caudal vein plexus (CVP) and intersegmental vessels (ISV), vascular defects, pericardial edema, circulation defect, and aberrant somite. Collectively, these findings show that SH3GLB1 activity is essential in the auditory, cardiovascular, and muscular systems, where defective mitochondria play a significant role in the pathogenesis of associated diseases.

Transient transfection of EGFP-labeled full-length SH3GLB1 protein for HEK293T cells validated the specificity of the antibody. The postnatal expression of Sh3glb1 in the mouse cochlea was tracked by localizing Sh3glb1 protein at many time points (postnatal day 1/P1, P7, P14, P21, P35, P56, and P180) (Fig. S1A). Sh3glb1 protein expression decreased with age throughout early development (Fig. S1B). Before P56, Sh3glb1 expression in the mouse cochlea was mainly localized to the outer hair cells (OHCs), IHCs, and SGNs. By P180, expression was mainly limited to IHCs and SGNs (Fig. 1A).

**Figure 1:**
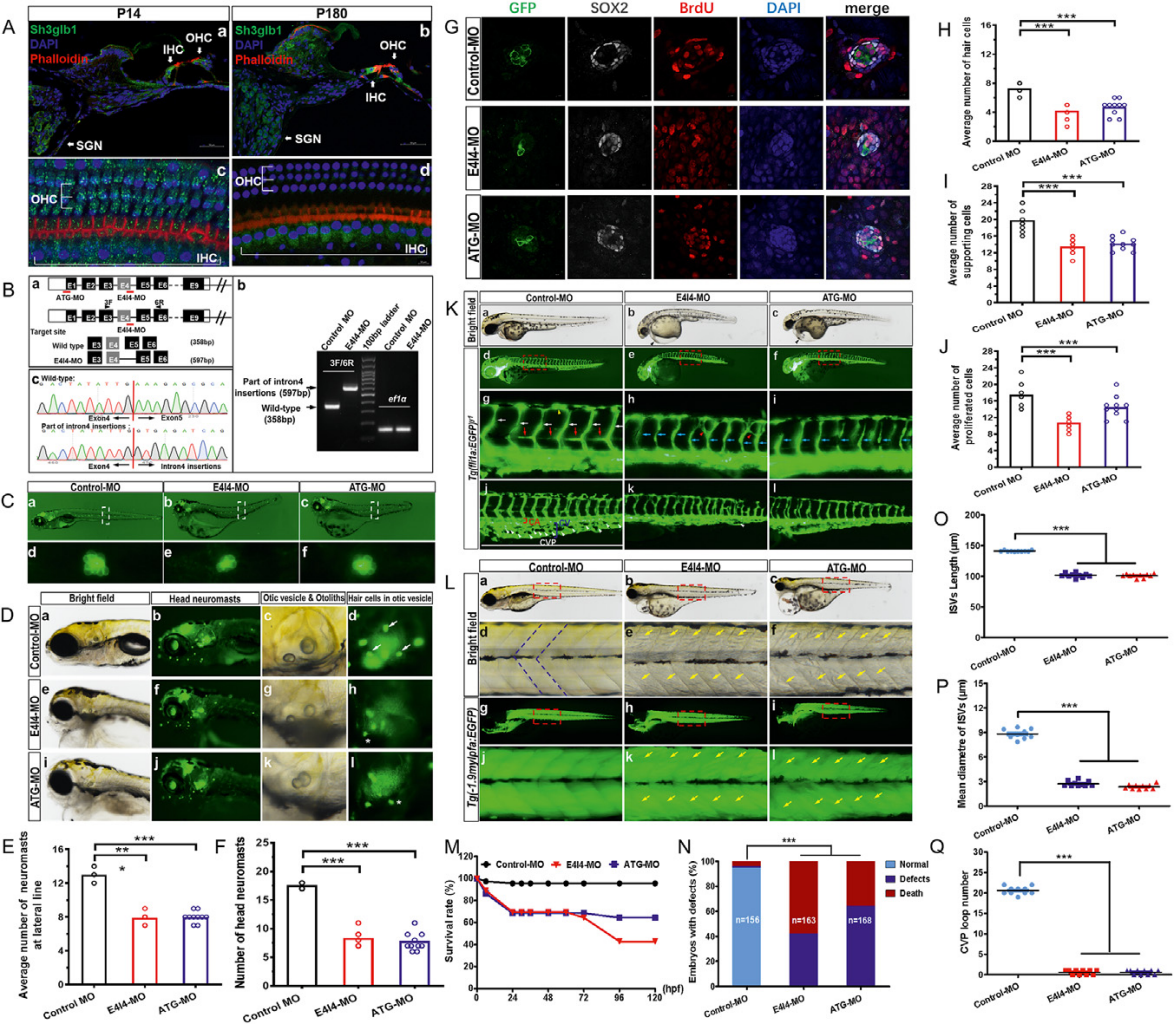
Immunofluorescence analysis of Sh3glb1 in mouse inner ear and *sh3glb1a*-knockdown analysis in the zebrafish. **(A)** Immunofluorescence analysis of Sh3glb1 localization at P14 and P180 mouse cochlea. **(B)** Design of zebrafish *sh3glb1a* morpholino. (a) The zebrafish *sh3glb1a* gene was targeted by specific MO antisense to prevent the appropriate splicing of exon 4 (E4I4-MO) and translation (ATG-MO). Primers 3F and 6R were designed to detect the presence of WT (non-mutant) transcripts or those in which exon 4 had been skipped. Schematic depiction of precursor mRNA in the E4I4-MO-injected embryos compared with control embryos is as shown below. (b) Electropherograms of wild-type and intron 4 insertions. (c) Sanger sequencing of both the wild type band and the intron 4-inserted band validating the wild type sequence and the intron 4-inserted sequence. **(C)** Lateral view of *sh3glb1a* zebrafish morphants. (a–c) The 7 dpf live Brn3c:mGFP transgenic embryo showing GFP expression by RGCs (retinal ganglion cells) and neuromasts (green dots) of the lateral line and head. (d–f) Control zebrafish exhibited normal hair cell numbers. In contrast, significantly decreased hair cells of the lateral line were observed in *sh3glb1a* morphants. The white boxed regions are shown at higher magnification in the below panels. **(D)** Gross head neuromasts and inner ear morphology of Tg(Brn3c:mGFP) embryos at 7 dpf. Compared with control MO (a–d), *sh3glb1a* deficiency caused head neuromast loss (f, j), small otoliths (g, k), and hair cell damage in otic vesicles (h, l, asterisk). dpf, days post fertilization. **(E)** Quantification of the average number of neuromasts at the lateral line. Scatter plot with bars; ^∗^*P* < 0.05, ^∗∗^*P* < 0.01, ^∗∗∗^*P* < 0.001 (*n* = 10; ANOVA). **(F)** Quantification of the average number of neuromasts at the head. Scatter plot with bars; ^∗∗∗^*P* < 0.001 (*n* = 10; ANOVA). **(G)** There were fewer hair cells, supporting cells, and proliferative cells in *sh3glb1a* morphants than in WT. BrdU, bromodeoxyuridine; DAPI, 2-(4-(dimethylamino)styryl)-N-ethylpyridinium iodide. **(H–J)** Quantifying the average number of hair cells, supporting cells and proliferative cells showed a significant decrease in *sh3glb1a* morphants. Scatter plot with bars; ^∗∗∗^*P* < 0.001 (*n* = 10; ANOVA). **(K)** Representative bright field and fluorescent images of *Tg(fli1a: EGFP)*^*y1*^ embryos at 2 dpf. The red boxed regions in (d-f) are shown at higher magnification in the below panels (g-i). Image of trunk regions taken at 2 dpf, with the vascular structures visualized by EGFP fluorescence and labeled ISV (intersegmental vessel) and DLAV (dorsal longitudinal anastomotic vessel) showed regular development in the embryo injected with control MO (d, g). Compared with control MO, the *sh3glb1a* morphant led to pericardial edema (b, c, black arrowheads), thinner ISVs (h, i, blue arrows), and ectopic branches between individual segmental arteries (h, red arrowheads). In control embryos, the parachordal vessels (PAV) normally formed (g, red arrows). Compared with the control, *sh3glb1a* morphant prevented the parachordal vessel (PAV) formation (h, i), the precursor to the lymphatic system. In control embryos, caudal vein plexus (CVP) formed honeycomb-like structures at the tail around 2 dpf (j, white arrowheads). In contrast, *sh3glb1a* morphant resulted in specific defects in CVP formation (k, l). Yellow arrowheads, dorsal longitudinal anastomotic vessels (DLAV); white arrows, normal ISVs; CA, caudal artery, CV, caudal vein. (L) Gross somite morphology at 4 dpf. The red boxed regions in (a-c) and (g-i) are shown at higher magnification in the below panels (d-f) and (j-l), respectively. Compared with control MO (d, V-shape somites), *sh3glb1a* morphant led to abnormal somites (e, f, k, l, yellow arrows). Blue dotted lines delineate the somite boundary. **(M)** A time-course plot of survival rate in control *vs*. *sh3glb1a* morphants for 120 hpf. hpf, hours post fertilization. **(N)** The percentage of embryos with development defects in control *vs*. *sh3glb1a* morphants at 5 dpf. dpf, days post fertilization. **(O, P)** Quantifying the length or mean diameter of intersegmental veins (ISVs) shows a significant decrease in *sh3glb1a*-MO injected embryos at 4 dpf. SEM (*n* = 10; ANOVA), ^∗∗∗^*P* < 0.001. dpf, days post fertilization. **(Q)** Quantification of loop number at caudal vein plexus (CVP) shows a significant decrease in *sh3glb1a*-MO injected embryos at 4 dpf. SEM (*n* = 10; ANOVA), ^∗∗∗^*P* < 0.001.

RT-PCR confirmed *sh3glb1a* expression in the zebrafish at various time points before 6 dpf (days post fertilization). After 6 hpf (hours post fertilization), the mRNA level of sh3glb1a decreased significantly, in accordance with the expression pattern observed in the mouse inner ear, indicating its role in the early development of zebrafish and auditory system (Fig. S1C). By utilizing AB line zebrafish and Tg (Brn3c:mGFP) s356t transgenic zebrafish (provided by Prof. Huawei Li, Fudan University), we built *sh3glb1a* morpholino-modified antisense oligonucleotide (MO) knockdown zebrafish throughmicroinjection of antisense MO into fertilized, one-cell embryos. Table S1 provides a list of the MO sequences. Two morphants were generated (E4I4-MO and ATG-MO) and the knockdown effectiveness of *sh3glb1a* was confirmed by RT-PCR (Fig. 1B). Compared with normal controls, both morphants had a reduced number of neuromasts at the lateral line and head (Fig. 1C, D). The average number of lateral line neuromasts in 7 dpf zebrafish was 7.9 ± 0.57 for E4I4-MO, 8 ± 0.67 for ATG-MO, and 13 ± 0.67 for WT, and that of head neuromasts in 7 dpf zebrafish was 8.2 ± 1.32 for E4I4-MO, 7.7 ± 1.58 for ATG-MO, and 17.4 ± 0.52 for WT (Fig. 1E, F). Using four markers, namely, SOX2 (supporting cells), GFP (hair cells), BrdU (proliferative cells), and DAPI (nucleus), we quantified the number of supporting cells and hair cells per neuromast in 6 dpf Tg (Brn3c:mGFP) transgenic zebrafish larvae. Neuromasts in the lateral line of MO knockdown larvae had fewer supporting cells and hair cells per neuromast than WT (Fig. 1G). The average number of hair cells per neuromast in 6 dpf zebrafish was 4.2 ± 1.03 for E4I4-MO, 4.7 ± 1.06 for ATG-MO, and 7.1 ± 0.88 for WT. The average number of supporting cells per neuromast was 13.2 ± 1.62 for E4I4-MO, 14.2 ± 1.62 for ATG-MO, and 19.5 ± 2.32 for WT (Fig. 1H, I). We exposed 4 dpf larvae to BrdU for 48 h and then used immunolabeling to observe cell proliferation in wild-type (WT) and morphant (MO) animals to evaluate the loss of IHCs and supporting cells associated with reduced cell proliferation. MOs had a much lower percentage of BrdU-positive cells than wild-type mice. The average number of proliferative cells per neuromast was 10.8 ± 1.69 for E4I4-MO, 14.5 ± 2.68 for ATG-MO, and 17.5 ± 2.88 for WT (Fig. 1J). In the otic vesicle, *sh3glb1a* deficiency causes hair cell damage and small otoliths. (Fig. 1D, asterisk).

The development of blood vessels in zebrafish was examined by injecting *sh3glb1a*-MO and control-MO into one-cell *fli1a*-EGFP transgenic line embryos after fertilization, while the somitogenesis was assessed by injecting *sh3glb1a*-MO and control-MO into one-cell Tg (−1.9mylpfa: EGFP) embryos. Aberrant *sh3glb1a* function induced vascular defects, pericardial edema, defective circulation, and abnormal somitogenesis. The phenotypes of pericardial edema, circulation defects, and abnormal somites in *sh3glb1a*-E4I4 and *sh3glb1a*-ATG morphants were nearly identical (Fig. 1K, L; Movie S1–6), and both morphants had significantly lower survival rates than controls (41.72% and 64.29% in E4I4-MO and ATG-MO, respectively; *n* = 163 and 168 embryos, respectively) (Fig. 1M, N). The two most prominent vascular patterns seen during zebrafish embryonic angiogenesis were ISVs and CVPs (Fig. 1K). ISV development deficit and mis-pattern plexus at CVP were seen in *sh3glb1a* mutants (Movie S4–6). ISV growth slowed significantly in the mid-somite region at 48 hpf (Fig. 1Kg–i). The mean ISV length of E4I4-MO is 101.5 μm (100.8, ATG-MO), compared with 141.2 μm in controls (*n* = 10). The mean diameter of ISV length of E4I4-MO is 2.725 μm (2.349, ATG-MO), compared with 8.817 μm in controls (*n* = 10) (Fig. 1O, P). The second trait we noticed at 48 hpf was the destruction of the honeycomb structure in the CVP compared to controls (Fig. 1Kj–l). At 48 hpf, loop formation was measured quantitatively and found to be reduced by a factor of 30 in *sh3glb1a* morphants (*n* = 10) (Fig. 1Q). According to our results, sh3glb1a controls ISV and CVP development during angiogenesis. As a result of poor blood vessel development, patients often have secondary complications such as edema and poor blood circulation. Pericardial edema, circulation abnormalities, and abnormally slow heart rates were observed when the expression of *sh3glb1a* was reduced (Fig. 1Ka–c; Movie S1–3). These findings support vascular abnormalities in *sh3glb1a* embryos. Meanwhile, muscle fibers in myotomes in both morphants appeared as a less compact and orderly-looking arrangement (Fig. 1L), indicating a disrupted somite development pattern which may be related to mitochondrial dysfunction or associated with highly conserved signaling pathways, such as FGF and Wnt.^3^

The following is/are the supplementary data related to this article.

Movie S1 Heartbeat is visible in the control zebrafish. Movie S1

Movie S2 Heartbeat is abnormal in *sh3glb1a*-E4I4-MO zebrafish. Movie S2

Movie S3 Heartbeat is abnormal in *sh3glb1a*-ATG-MO zebrafish. Movie S3

Movie S4 Circulation in the caudal vein (CV) is visible in the control zebrafish. Movie S4

Movie S5 Circulation in the caudal vein (CV) is abnormal in *sh3glb1a*-E4I4-MO zebrafish. Movie S5

Movie S6 Circulation in the caudal vein (CV) is abnormal in *sh3glb1a*-ATG-MO zebrafish. Movie S6

In this work, we examined the localization of Sh3glb1 in the inner ear of mice and generated two morpholino knockdown zebrafish models to examine the possible function of sh3glb1 in auditory and other systems. We observed hair cell loss in zebrafish after *sh3glb1* was knocked down, indicating that normal levels of sh3glb1 play a role in controlling inner ear development in zebrafish. Defects in other tissues, including circulatory and muscular systems, were also seen in the morphants at an early stage of development, consistent with the presentation of mitochondrial disease.^4^^,^^5^ These findings suggested that abnormal sh3glb1 might trigger imbalanced mitophagy and contribute to developing several symptoms of illnesses involving the mitochondria. More functional research is required to determine whether inner ear structures are compromised in mice with aberrant Sh3glb1 and if these animals experience hearing loss.

## Ethics declaration

This study was approved by the Chinese People's Liberation Army General Hospital Research Ethics Committee (No. S2016-120-02).

## Author contributions

Conceptualization, P.D., Y.Y.Y. and X.G.; Methodology, W.Q.W., K.Y., M.Y.H. Y.J.C. and D.Y.K.; Software, J.Y.Y; Data analysis and curation, S.S.H.; Writing-Original draft preparation, X.G. and W.Q.W.; Writing-review & editing, Y.Y.Y. and G.J.W.; Project administration, Z.D.W. All authors read and approved the final manuscript.

## Conflict of interests

The authors declare no conflict of interests.

## Funding

This research was supported by grants from the 10.13039/501100001809National Natural Science Foundation of China (No. 82171158, 82271177, 82271185, 82171155) and the National Key Research and Development Project of China (No. 2020YFC2008500). The funders had no role in study design, data collection and analysis, decision to publish, or preparation of the manuscript.

## Data availability

The data presented in this study are available on request from the corresponding author.

## Figures and Tables

**Fig. S1:**
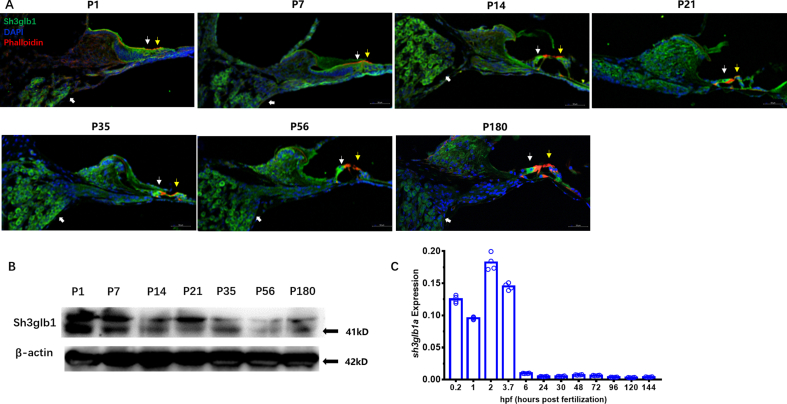
Expression of Sh3glb1/sh3glb1a in mouse cochlea/zebrafish at different stages of development. **(A)** Immunofluorescence analysis of Sh3glb1 in mouse cochlea at different stages of development (P1, P7, P14, P21, P35, P56, and P180). Sh3glb1 (green) localized in SGNs (thick white arrows), IHCs (thin white arrows), and OHCs (thin yellow arrows). **(B)** Western blot analysis of Sh3glb1 in mouse cochlea at different stages of development (P1, P7, P14, P21, P35, P56, and P180). **(C)** qRT-PCR for twelve embryo development stages (0.2 hpf, 1 hpf, 2 hpf, 3.7 hpf, 6 hpf, 24 hpf, 30hpf, 48 hpf, 72 hpf, 96 hpf, 120 hpf, and 144 hpf) demonstrate different expression levels of <i>sh3glb1a </i>during embryonic development. hpf, hours post fertilization.

## References

[1] Zhang Y., Fang Q., Wang H. (2023). Increased mitophagy protects cochlear hair cells from aminoglycoside-induced damage. Autophagy.

[2] Wang D.B., Kinoshita Y., Kinoshita C. (2015). Loss of endophilin-B1 exacerbates Alzheimer's disease pathology. Brain.

[3] Heilig A.K., Nakamura R., Shimada A. (2022). Wnt 11 acts on dermomyotome cells to guide epaxial myotome morphogenesis. Elife.

[4] Kumar A.A., Kelly D.P., Chirinos J.A. (2019). Mitochondrial dysfunction in heart failure with preserved ejection fraction. Circulation.

[5] Chen T.H., Koh K.Y., Lin K.M.C., Chou C.K. (2022). Mitochondrial dysfunction as an underlying cause of skeletal muscle disorders. Int J Mol Sci.

