# Supplementary material for: Characterization of SH3GLB1 in the auditory system and its potential role in mitophagy
Source: Genes Dis. 2023 Jul 6;11(4):101018. doi: 10.1016/j.gendis.2023.05.017 (PMC10940771; doi:10.1016/j.gendis.2023.05.017)
Supplement: Multimedia component 1 [file mmc1.docx]

**Table S1 Morpholino sequences**

| **Morpholino Name** | **Morpholino oligo sequence** |
| --- | --- |
| *sh3glb1a*-E4I4-MO (E4I4-MO) | 5’-GCTGCCTGATCTCACCAATATAGTC-3’ |
| *sh3glb1a*-ATG-MO (ATG-MO) | 5’-ATCTCTTCACGTTAAAGTCCATCTT-3’ |
| Standard Control MO | 5’-CCTCTTACCTCAGTTACAATTTATA-3’ |
