# Supplementary material for: Characterization of SH3GLB1 in the auditory system and its potential role in mitophagy
Source: Genes Dis. 2023 Jul 6;11(4):101018. doi: 10.1016/j.gendis.2023.05.017 (PMC10940771; doi:10.1016/j.gendis.2023.05.017)
Supplement: Multimedia component 8 [file mmc8.docx]

**Materials and Methods**

**1 Localization of Sh3glb1 in the mouse inner ear**

To ensure the reliability of the observed expression patterns, three sets of slides covering the whole inner ear were stained from three separate mice at 1, 7, 14, 21, 35, 56, and 180 days of age. C57 mouse ears were fixed in 4% paraformaldehyde (PFA), decalcified, and dehydrated through a succession of ethanol concentrations before being cryosectioned into 8mm thick serial slices. Actin was stained with Phalloidin-iFluor 555 (1:1000, Abcam, Cambridge, UK), and the anti-Sh3glb1 rabbit polyclonal antibody was diluted (1:200, Atlas, Sweden). The nuclei were stained with DAPI (1×, ZSJB-Bio, Beijing, China), and the primary antibody was omitted as a control. Confocal microscopy was used to analyze sections. Mouse cochlea ducts were dissected, fixed, treated with anti-Sh3glb1 (1:200) and DAPI, and examined under a confocal microscope (Zeiss) for the whole-mount organ of Corti immunostaining.

**2 RNA extraction and quantitative real-time PCR (qRT-PCR)**

Using Trizol (Roche, IN, USA), total RNA was isolated from 30-50 zebrafish embryos per group as recommended. RNA was reverse transcribed utilizing a PrimeScript RT reagent Kit that included a gDNA Eraser (Takara, Otsu, Japan). Gene expression levels were measured in triplicate using Bio-Rad iQ SYBR Green Supermix (Bio-Rad, Hercules, CA) and the Realplex system for detection (Eppendorf). Using *ef1α* as an endogenous control gene, we used the comparative threshold cycle approach (2 - ΔΔCt) to calculate relative gene expression levels. All primer sequences are shown in Table S1.

**3 Cell culture and transfection**

Cells from the human embryonic kidney (HEK) 293T were cultured in six-well plates with DMEM Medium (C11965500BT, Gibco) supplemented with 10% fetal bovine serum (10090, Gibco) and 1% Penicillin Streptomycin (15140-122, Gibco). At a density of 60-80%, we transfected the cells using pEGFP-N1-CMV-*SH3GLB1* (encoding full-length of human SH3GLB1, NM 001206651)-EGFP. 1µg of plasmid and 2 µl of lipofectamine 2000 (2150081, Invitrogen) diluted in 125 µl of Opti-MEM (31985-070, Gibco) were used for transfection. After 24 hours, cells were fixed in 4% PFA and permeabilized with 0.2% Triton-X100. Cells were treated with Rabbit anti-Sh3glb1 antibody (HPA015608, Atlas) at 4 °C overnight after a blocking step with 4% goat serum. After three washes in PBS, cells were incubated at room temperature for two hours with Alexa Fluor 568 goat anti-rabbit IgG (A-11036, Thermo Fisher Scientific). DAPI was used to stain the nuclei. An inverted confocal microscope (LSM 780) made by Zeiss was used to capture the pictures.

**4 Zebrafish husbandry and** ***sh3glb1a* morpholino-modified antisense oligonucleotides (MO) knockdown**

AB line zebrafish and Tg (Brn3c:mGFP) s356t transgenic zebrafish (provided by Prof. Hua–Wei Li, Fudan University)  were utilized to produce GFP in hair cells. Microinjection of antisense MO into fertilized, one-cell embryos was performed following established procedures[[13](#_ENREF_13)]. Two different morpholino antisense techniques were used to silence the zebrafish *sh3glb1a* gene: one inhibited translation of the zebrafish gene (ATG-MO), and the other blocked exon4 splicing (E4I4–MO). Table S2 provides a list of the MO sequences. To verify the effectiveness of the E4I4-MO, RT-PCR analysis was performed using primers spanning exons 3 (forward primer: 5’-TGAGCTTATGGGTCAGTCTAT-3') and 6 (reverse primer: 5’-CAGAAGAAGCCTGGTGATTT-3') of *sh3glb1a*.

**5 Cell proliferation and immunofluorescence staining assays in zebrafish**

4-dpf (days post fertilization) MO and WT (Wild-type) larvae were exposed to 15 mM bromodeoxyuridine (BrdU; Sigma-Aldrich) in EM for 48 h at 28.5 °C. After being washed three times in PBST containing 0.1% TritonX-100, they were fixed in 4% PFA overnight at 4 °C or for 4 hours at room temperature (RT). Each larva was exposed to 2 N HCl at RT for 1 hour. We repeated the PBST washing and blocked the larvae for 1 hour at RT with 10% normal goat serum in PBST. The larvae were incubated with the primary antibody, including the rabbit monoclonal anti-sox2 (1:200, Abcam, ab137385), Chicken anti-GFP (1:200, Abcam, ab13970), mouse anti-BrdU IgG (1:200, Santa Cruz Biotechnology, TX, USA) overnight at 4°C. The secondary antibodies including Cy3 anti-Mouse (1:200, Jackson ImmunoResearch Laboratories, USA), Alexa Fluor 488 anti-Chicken (1:200, Life Technologies, A11008), and Alexa Fluor 647 anti-Rabbit (1:200, Abcam, ab150075) were used at 4°C overnight. DAPI was applied for 20 minutes while at RT. Using a confocal microscope, cell counts were taken.

**6 Angiogenesis evaluation**

The development of blood vessels in zebrafish was examined by injecting *sh3glb1a*-MO and control-MO into one-cell *fli1a*-EGFP transgenic lines embryos after fertilization. Embryos were anesthetized with 0.016% MS-222 after they were dechorionated 2 days after fertilization (tricaine methane sulfonate, Sigma-Aldrich). Before being mounted with 3% methylcellulose on a depression slide, zebrafish were orientated laterally (posterior to the right, anterior to the left, and dorsal to the top) for viewing with fluorescence microscopy. Quantitative analyses were performed on the phenotypes of the CVP and ISVs.

**7 Zebrafish somitogenesis studies**

One-cell Tg (-1.9mylpfa: EGFP) embryos were injected with either *sh3glb1a*-MO or control-MO after fertilization to assess somitogenesis in zebrafish. As early as 4-dpf, embryos were dechorionated to render them unconscious. Somatic phenotypes at the trunk of the body were examined.

**8 Image acquisition and statistical analysis**

The Nikon SMZ 18 fluorescent microscope was used to examine the embryos and larvae, and then digital cameras were used to capture images of the specimens. Image-based morphometric analysis (NIS-Elements D4.6, Japan) and ImageJ (NIH, USA) were used to perform the quantitative image analyses. For this analysis, we flipped fluorescent photos upside down. Using ImageJ, we determined that a certain amount of particles represented a positive signal. The average signal strength across ten animals in each treatment group was calculated. All data are presented as mean ± SEM. GraphPad Prism 5.0 (GraphPad Software, CA) was used for statistical analysis and graphical data display. Student's t-test, analysis of variance (ANOVA), and χ2 test were used to determine statistical significance. Statistical significance is indicated by *, where P < 0.05, and ***, where P < 0.001.
